# Supplementary material for: Longitudinal analysis of the prevalence of minor Plasmodium spp. in the reservoir of asymptomatic infections through sequential interventions in Northern Sahelian Ghana
Source: medRxiv. 2025 Aug 19:2025.08.15.25333797. Preprint. [Version 1] doi: 10.1101/2025.08.15.25333797 (PMC12393655; doi:10.1101/2025.08.15.25333797)
Supplement: 1 — S1 Table. Parasitological characteristics of the Plasmodium spp. infections (including single- and mixed-species infections) during each study time point. S2 Table. Association between the study time points and P. malariae and P. ovale spp. infection prevalence using the species-specific 18S rRNA PCR. S3 Table. Stratum-specific estimates for the association between age groups and P. malariae prevalence during each study time point. S4 Table. Stratum-specific estimates for the association between age and P. ovale spp. prevalence during each study time point. Fig S1. Proportion of single- and mixed-Plasmodium spp. infections identified using the species-specific 18S rRNA PCR during each study time point. Proportion of single- and mixed-species infections (A-C) in the population and (D-F) by age group for P. falciparum (green), P. malariae (blue), and P. ovale spp. (red) during each study time point. For each Plasmodium spp. darker shades represent those isolates with single-species infections, while the lighter shades denote those isolates with mixed-species infection (including double- and triple-species infections). Blank or white spaces indicate no infections detected by the species-specific 18S rRNA PCR. [file NIHPP2025.08.15.25333797V1-supplement-1.pdf]

medRxiv preprint doi: https://doi.org/10.1101/2025.08.15.25333797; this version posted August 19, 2025. The copyright holder for this preprint (which was not certified by peer review) is the author/funder, who has granted medRxiv a license to display the preprint in perpetuity.
It is made available under a CC-BY 4.0 International license .

S1 Table. Parasitological characteristics of the *Plasmodium* spp. infections (including single- and mixed-species infections) during each study time point.

**Parasitological parameters**	**October 2012** (Survey 1, pre-IRS)	**October 2015** [a] (Survey 2, post-IRS)	**October 2017** (Survey 3, SMC)	**November 2020** (Survey 4, SMC)	**October 2022** (Survey 5, SMC)
**Number of DBS tested**	1,923	2,020	1,915	1,809	2,026
***P. falciparum* prevalence** (including single- and mixed-species infections)					
**All** [b]	**1,402 (72.9)**	**788 (39.0)**	**1,214 (63.4)**	**896 (49.5)**	**1,036 (51.1)**
**Age groups** [c]					
< 5 years	186 (68.6)	66 (20.6)	70 (23.7)	42 (12.9)	86 (21.9)
5-10 years	398 (82.9)	244 (49.5)	268 (64.3)	152 (46.6)	202 (45.4)
11-20 years	344 (83.3)	251 (53.9)	414 (84.7)	356 (69.1)	366 (71.3)
≥ 21 years	474 (62.5)	227 (30.6)	462 (64.7)	346 (53.9)	382 (56.6)
**Sex** [c]					
Female	711 (69.0)	389 (35.6)	646 (62.4)	490 (49.7)	543 (48.5)
Male	691 (77.5)	399 (43.0)	568 (64.6)	406 (49.3)	493 (54.4)
**Catchment area** [c]					
Soe	801 (79.8)	440 (43.1)	685 (68.3)	478 (51.1)	528 (50.9)
Vea/Gowrie	601 (65.4)	348 (34.8)	529 (58.0)	418 (47.8)	508 (51.4)
***P. malariae* prevalence** (including single- and mixed-species infections)					
**All** [b]	**264 (13.7)**	**29 (1.4)**	**55 (2.9)**	**133 (7.4)**	**117 (5.8)**
**Age groups** [c]					
< 5 years	28 (10.3)	0 (0.0)	1 (0.3)	0 (0.0)	1 (0.3)
6-10 years	134 (27.9)	6 (1.2)	14 (3.4)	21 (6.4)	18 (4.0)
11-20 years	61 (14.8)	13 (2.8)	29 (5.9)	99 (19.2)	75 (14.6)
≥ 21 years	41 (5.4)	10 (1.3)	11 (1.5)	13 (2.0)	23 (3.4)
**Sex** [c]					
Female	123 (11.9)	13 (1.2)	25 (2.4)	56 (5.7)	42 (3.8)
Male	141 (15.8)	16 (1.7)	30 (3.4)	77 (9.4)	75 (8.3)
**Catchment area** [c]					
Soe	166 (16.5)	19 (1.9)	42 (4.2)	70 (7.5)	50 (4.8)
Vea/Gowrie	98 (10.7)	10 (1.0)	13 (1.4)	63 (7.2)	67 (6.8)
***P. ovale* spp. prevalence** (including single- and mixed-species infections)					
**All** [b]	**110 (5.7)**	**8 (0.4)**	**76 (4.0)**	**47 (2.6)**	**26 (1.3)**
**Age groups** [c]					
< 5 years	8 (3.0)	0 (0.0)	1 (0.3)	0 (0.0)	0 (0.0)
6-10 years	34 (7.1)	1 (0.2)	22 (5.3)	8 (2.5)	5 (1.1)
11-20 years	39 (9.4)	5 (1.1)	39 (8.0)	28 (5.4)	19 (3.7)
≥ 21 years	29 (3.8)	2 (0.3)	14 (2.0)	11 (1.7)	2 (0.3)
**Sex** [c]					
Female	52 (5.0)	3 (0.3)	32 (3.1)	18 (1.8)	11 (1.0)
Male	58 (6.5)	5 (0.5)	44 (5.0)	29 (3.5)	15 (1.7)
**Catchment area** [c]					
Soe	66 (6.6)	7 (0.7)	57 (5.7)	24 (2.6)	15 (1.4)
Vea/Gowrie	44 (4.8)	1 (0.1)	19 (2.1)	23 (2.6)	11 (1.1)

[a] For the *Plasmodium* spp. infections in October 2015, there were 2,020 participants included in the analysis: participants were excluded when no DBS was available for PCR (N=2).

[b] Data reflects the number (% (n/N)) of participants sampled that were positive for *P. falciparum, P. malariae,* or *P.ovale* spp. (including single- and mixed-species infections) as determined by *18S rRNA* PCR, relative to the total number of DBS samples tested. See Table 2 for the breakdown of the *Plasmodium* spp. groupings for single and mixed infections.

[c] Data reflects the number (% (n/N)) of participants sampled that were positive for *P. falciparum, P. malariae,* or *P. ovale* spp. (including single- and mixed-species infections) as determined by *18S rRNA* PCR, relative to the total number of DBS samples tested for each age group, sex, and catchment area. See Table 1 for the demographic breakdown by age group, sex, and catchment area.

S2 Table. Association between the study time points and *P. malariae* and *P. ovale* spp. infection prevalence using the species-specific *18S rRNA* PCR.

	P. malariae **infection** (including single- and mixed-species infection) [a]				***P. ovale*** **spp. infection** (including single- and mixed-species infection) [a]			
	Unadjusted		Adjusted [b]		Unadjusted		Adjusted [b]	
**Factor**	OR (95% CI)	*p*-value	aOR (95% CI)	*p*-value	OR (95% CI)	*p*-value	aOR (95% CI)	*p*-value
**Study time point (Survey, Intervention)**								
October 2012 (Survey 1, pre-IRS)	1.00	-	1.00	-	1.00	-	1.00	-
October 2015 (Survey 2, post-IRS)	0.10 (0.07-0.14)	**<0.001**	0.08 (0.05-0.11)	**<0.001**	0.07 (0.03-0.14)	**<0.001**	0.06 (0.03-0.13)	**<0.001**
October 2017 (Survey 3, SMC)	0.18 (0.14-0.24)	**<0.001**	0.14 (0.11-0.19)	**<0.001**	0.70 (0.52-0.94)	**0.018**	0.62 (0.46-0.84)	**0.002**
November 2020 (Survey 4, SMC)	0.50 (0.41-0.62)	**<0.001**	0.42 (0.34-0.53)	**<0.001**	0.45 (0.31-0.63)	**<0.001**	0.40 (0.28-0.57)	**<0.001**
October 2022 (Survey 5, SMC)	0.39 (0.31-0.48)	**<0.001**	0.35 (0.27-0.44)	**<0.001**	0.21 (0.14-0.33)	**<0.001**	0.20 (0.13-0.31)	**<0.001**
**Age groups**								
< 5 years	1.00	-	1.00	-	1.00	-	1.00	-
5-10 years	5.13 (3.47-7.59)	**<0.001**	5.14 (3.50-7.57)	**<0.001**	5.92 (2.94-11.9)	**<0.001**	5.57 (2.74-11.3)	**<0.001**
11-20 years	6.77 (4.66-9.84)	**<0.001**	6.49 (4.46-9.44)	**<0.001**	10.1 (5.09-19.9)	**<0.001**	9.66 (4.84-19.3)	**<0.001**
≥ 21 years	1.48 (0.97-2.25)	0.067	1.36 (0.89-2.08)	0.2	2.99 (1.48-6.04)	**0.002**	2.81 (1.37-5.77)	**0.005**
**Sex**								
Female	1.00	-	1.00	-	1.00	-	1.00	-
Male	1.60 (1.33-1.92)	**<0.001**	1.39 (1.15-1.68)	**<0.001**	1.56 (1.22-1.99)	**<0.001**	1.41 (1.10-1.81)	**0.007**
**Catchment area**								
Soe	1.00	-	1.00	-	1.00	-	1.00	-
Vea/Gowrie	0.75 (0.62-0.90)	**0.002**	0.70 (0.58-0.85)	**<0.001**	0.60 (0.46-0.77)	**<0.001**	0.57 (0.44-0.74)	**<0.001**
**LLIN usage (previous night)**								
No	1.00	-	1.00	-	1.00	**-**	1.00	**-**
Yes	0.71 (0.54-0.92)	**0.011**	1.02 (0.77-1.36)	0.9	0.86 (0.56-1.30)	0.5	1.08 (0.70-1.68)	0.7
**Antimalarial treatment (previous two weeks)** [c]								
No treatment	1.00	-	1.00	-	1.00	-	1.00	**-**
Treatment	0.77 (0.63-0.94)	**0.011**	0.55 (0.44-0.68)	**<0.001**	0.84 (0.62-1.12)	0.2	0.76 (0.56-1.03)	0.076

OR = odds ratio; aOR = adjusted odds ratio; CI = confidence interval; LLIN = long-lasting insecticidal net

[a] Participants that were sick, sought treatment, but didn't know if they were provided with an antimalarial treatment in the previous two weeks were excluded from the model: October 2015 (N=79; 3.9%); October 2017 (N=44; 2.3%); November 2020 (N=26; 1.4%); and October 2022 (N=6; 0.3%).

[b] Age groups, sex, catchment area, LLIN usage (previous night), and antimalarial treatment (previous two weeks) are adjusted for in the multivariable logistic regression model.

[c] Indicates those participants who reported they were sick, sought treatment, and were provided with an antimalarial treatment in the previous two weeks. For those participants who didn't know if they were provided with an antimalarial treatment are coded as "Don't know".

S3 Table. Stratum-specific estimates for the association between age groups and *P. malariae* prevalence during each study time point.

	P. malariae **infection** (including single- and mixed-species infection) [a]								
	**October 2012 (Survey 1, pre-IRS)**	**October 2015 (Survey 2, post-IRS)**		**October 2017 (Survey 3, SMC)**		**November 2020 (Survey 4, SMC)**		**October 2022 (Survey 5, SMC)**	
**Factor**	aOR [b]	aOR (95% CI) [b]	*p-value*	aOR (95% CI) [b]	*p-value*	aOR (95% CI) [b]	*p-value*	aOR (95% CI) [b]	*p-value*
**Age groups**									
< 5 years	1.00	0.01 (0.00-0.08)	**<0.001**	0.04 (0.00-0.14)	**<0.001**	0.01 (0.00-0.07)	**<0.001**	0.03 (0.00-0.11)	**<0.001**
5-10 years	1.00	0.03 (0.01-0.06)	**<0.001**	0.07 (0.04-0.13)	**<0.001**	0.15 (0.09-0.25)	**<0.001**	0.10 (0.06-0.17)	**<0.001**
11-20 years	1.00	0.16 (0.08-0.29)	**<0.001**	0.35 (0.21-0.55)	**<0.001**	1.30 (0.92-1.86)	0.14	0.97 (0.68-1.41)	0.9
≥ 21years	1.00	0.24 (0.11-0.46)	**<0.001**	0.21 (0.10-0.42)	**<0.001**	0.35 (0.18-0.64)	**<0.001**	0.59 (0.35-1.00)	**0.048**

aOR= adjusted odds ratio; CI= confidence interval

[a] Participants that were sick, sought treatment, but didn't know if they were provided with an antimalarial treatment in the previous two weeks were excluded from the model: October 2015 (N=79; 3.9%); October 2017 (N=44; 2.3%); November 2020 (N=26; 1.4%); and October 2022 (N=6; 0.3%).

[b] Age groups, sex, catchment area, LLIN usage (previous night), and antimalarial treatment (previous two weeks) are adjusted for in the multivariable logistic regression model.

S4 Table. Stratum-specific estimates for the association between age and *P. ovale* spp. prevalence during each study time point.

	P. ovale **spp. infection** (including single- and mixed-species infection) [a]								
	**October 2012 (Survey 1, pre-IRS)**	**October 2015 (Survey 2, post-IRS)**		**October 2017 (Survey 3, SMC)**		**November 2020 (Survey 4, SMC)**		**October 2020 (Survey 5, SMC)**	
**Factor**	aOR [b]	aOR (95% CI) [b]	*p-value*	aOR (95% CI) [b]	*p-value*	aOR (95% CI) [b]	*p-value*	aOR (95% CI) [b]	*p-value*
**Age groups**									
< 5 years	1.00	0.04 (0.00-0.33)	**<0.001**	0.12 (0.01-0.58)	**0.007**	0.03 (0.00-0.29)	**<0.001**	0.03 (0.00-0.28)	**<0.001**
5-10 years	1.00	0.04 (0.00-0.14)	**<0.001**	0.67 (0.38-1.17)	0.2	0.33 (0.14-0.69)	**0.002**	0.16 (0.06-0.37)	**<0.001**
11-20 years	1.00	0.11 (0.04-0.25)	**<0.001**	0.78 (0.49-1.26)	0.3	0.53 (0.31-0.87)	**0.013**	0.36 (0.20-0.63)	**<0.001**
≥21 years	1.00	0.08 (0.02-0.26)	**<0.001**	0.51 (0.26-0.98)	**0.044**	0.45 (0.21-0.89)	**0.021**	0.09 (0.02-0.27)	**<0.001**

aOR= adjusted odds ratio; CI= confidence interval

[a] Participants that were sick, sought treatment, but didn't know if they were provided with an antimalarial treatment in the previous two weeks were excluded from the model: October 2015 (N=79; 3.9%); October 2017 (N=44; 2.3%); November 2020 (N=26; 1.4%); and October 2022 (N=6; 0.3%).

[b] Age groups, sex, catchment area, LLIN usage (previous night), and antimalarial treatment (previous two weeks) are adjusted for in the multivariable logistic regression model.

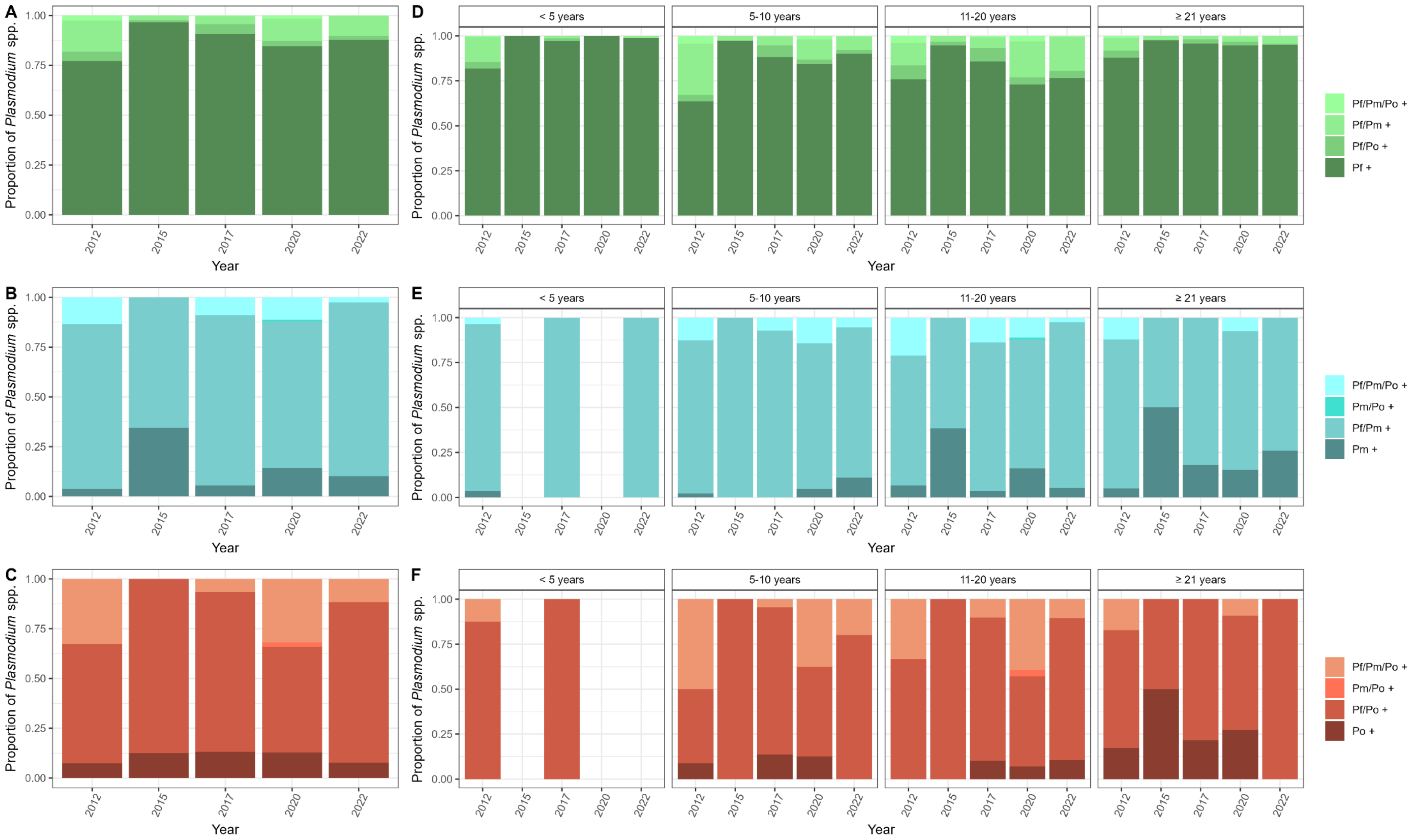

Fig S1. Proportion of single- and mixed-*Plasmodium* spp. infections identified using the species-specific *18S rRNA* PCR during each study time point. Proportion of single- and mixed-species infections (**A-C**) in the population and (**D-F**) by age group for *P. falciparum* (green)*, P. malariae* (blue), and *P. ovale* spp. (red) during each study time point. For each *Plasmodium* spp. darker shades represent those isolates with single-species infections, while the lighter shades denote those isolates with mixed-species infection (including double- and triple-species infections). Blank or white spaces indicate no infections detected by the species-specific *18S rRNA* PCR.
